# Supplementary material for: The interactome of intact mitochondria by cross-linking mass spectrometry provides evidence for coexisting respiratory supercomplexes
Source: Mol Cell Proteomics. 2017 Dec 8;17(2):216–32. doi: 10.1074/mcp.RA117.000470 (PMC5795388; doi:10.1074/mcp.RA117.000470)
Supplement: Supplemental Data [file supp_RA117.000470_133922_0_supp_23978_fzffwf.zip › spectra_annotation/mito_DR_spectra_annotation/241-1-6-1-7-1.pdf]

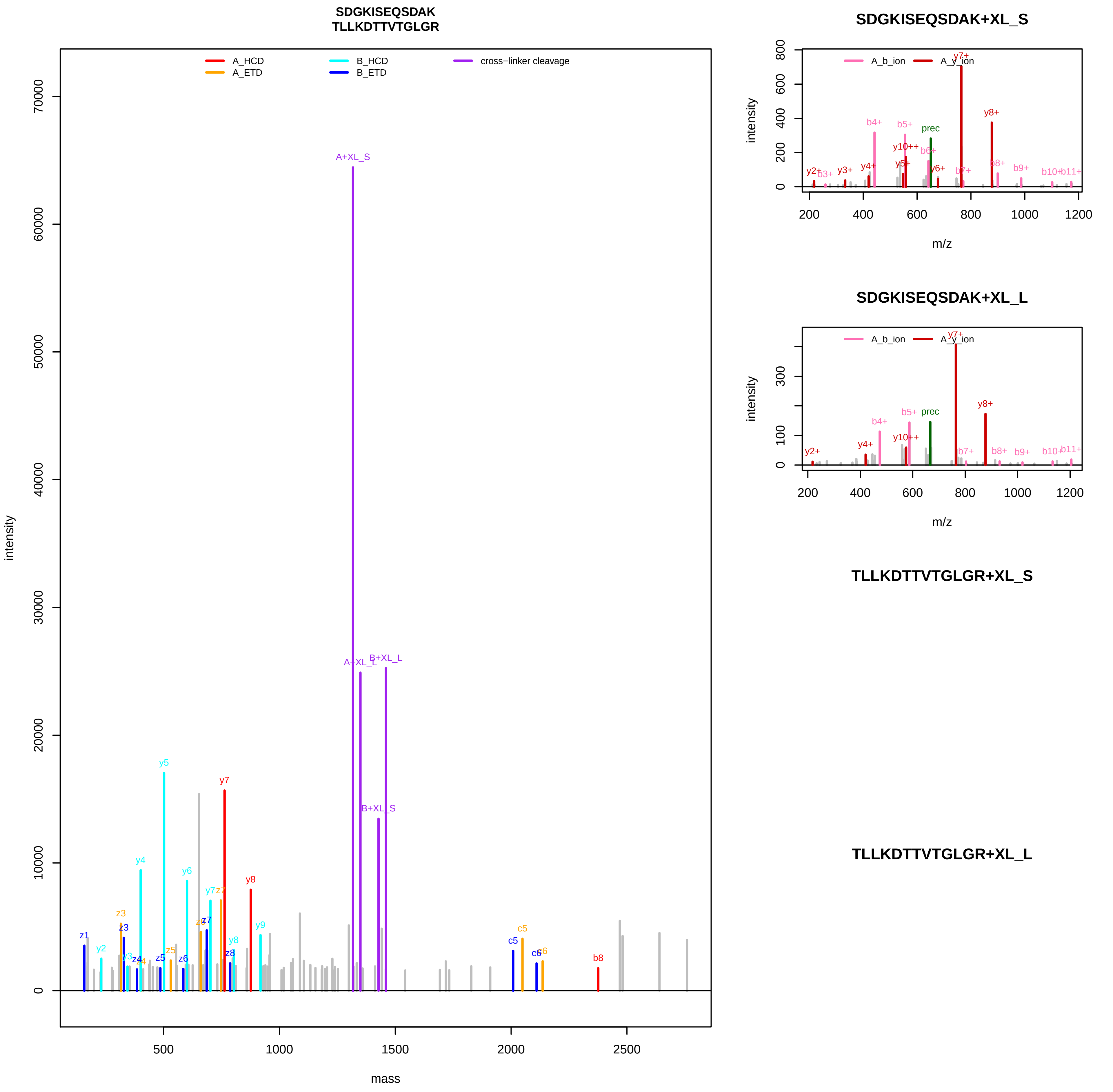
SDGKISEQSDAK
TLLKDTTVTGLGR
A_HCD
A_ETD
B_HCD
B_ETD
cross−linker cleavage
A+XL_S
A+XL_L
B+XL_L
B+XL_S
y2
y3
y4
y5
y6
y7
y8
y9
z1
z3
z4
z5
z6
z7
z8
c5
c6
y7
y8
b8
intensity
mass
0
10000
20000
30000
40000
50000
60000
70000
500
1000
1500
2000
2500
SDGKISEQSDAK+XL_S
A_b_ion
A_y_ion
y2+
b3+
y3+
y4+
b4+
b5+
y5+
y10++
prec
b6+
y6+
y7+
b7+
y8+
b8+
b9+
b10+
b11+
intensity
m/z
0
200
400
600
800
1000
1200
SDGKISEQSDAK+XL_L
A_b_ion
A_y_ion
y2+
y4+
b4+
y10++
b5+
prec
y7+
b7+
y8+
b8+
b9+
b10+
b11+
intensity
m/z
0
100
300
200
400
600
800
1000
1200
TLLKDTTVTGLGR+XL_S
TLLKDTTVTGLGR+XL_L
